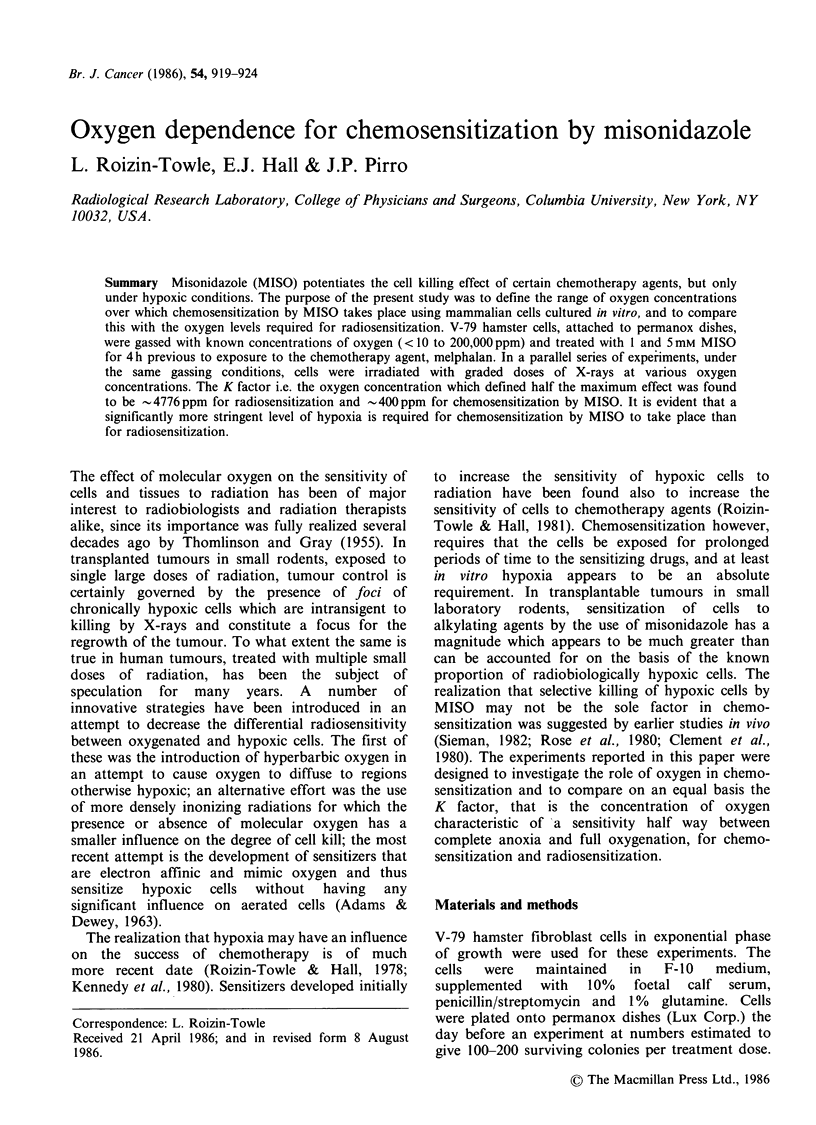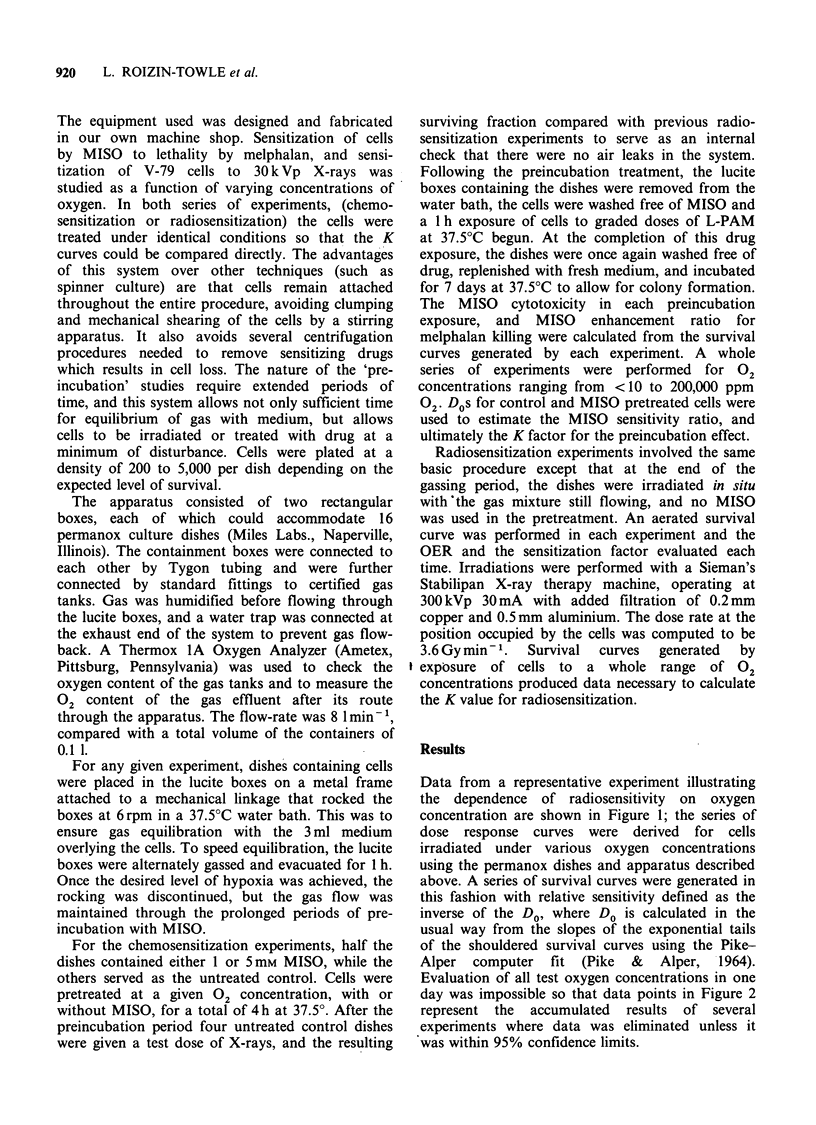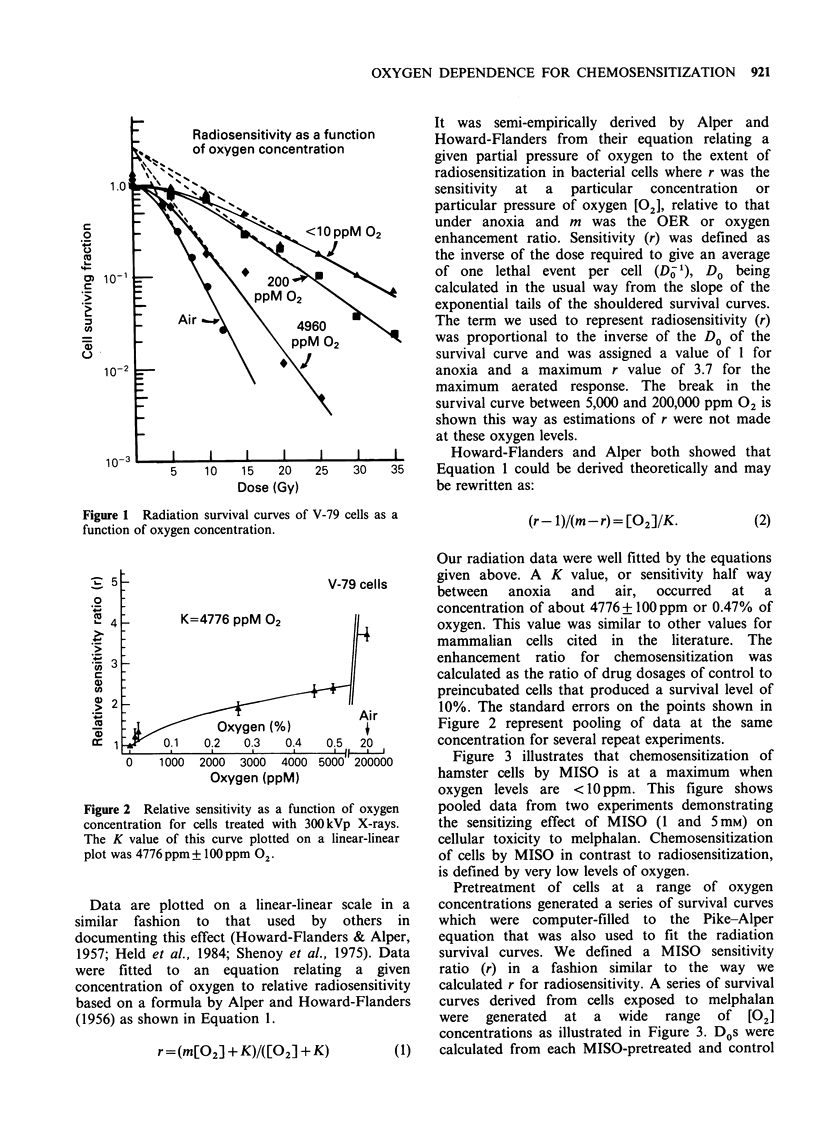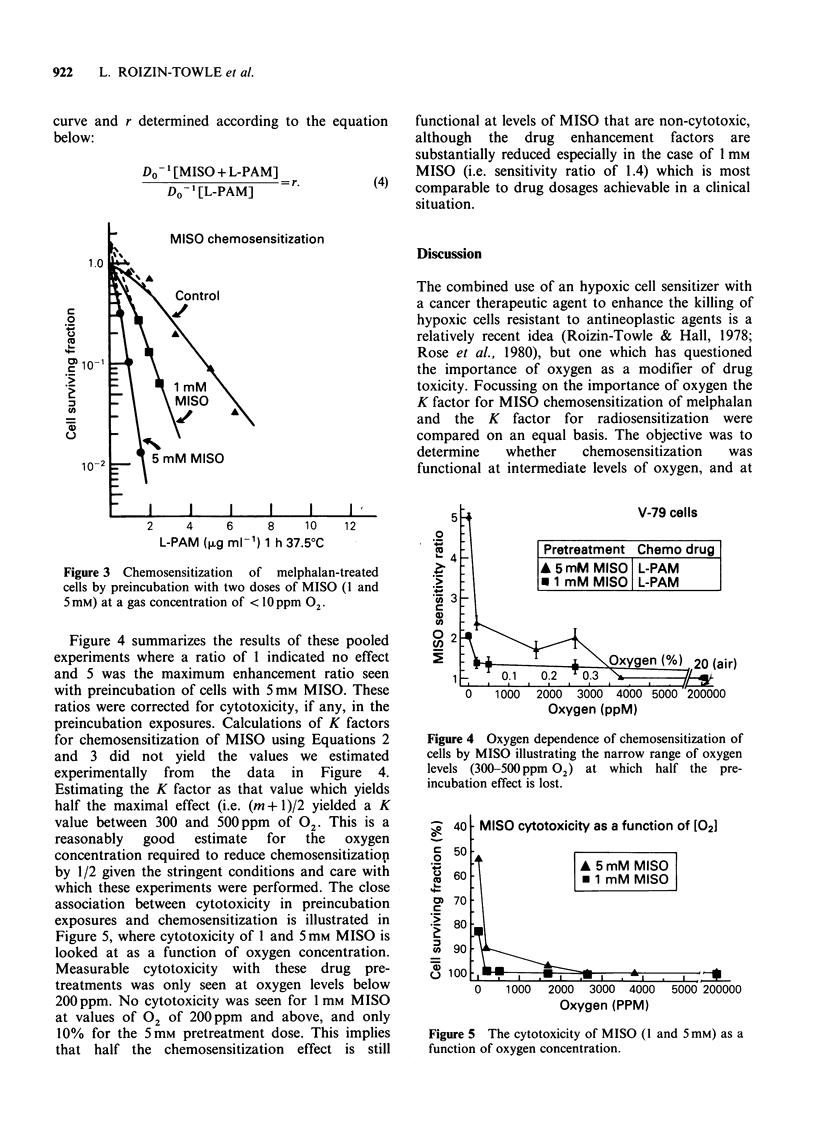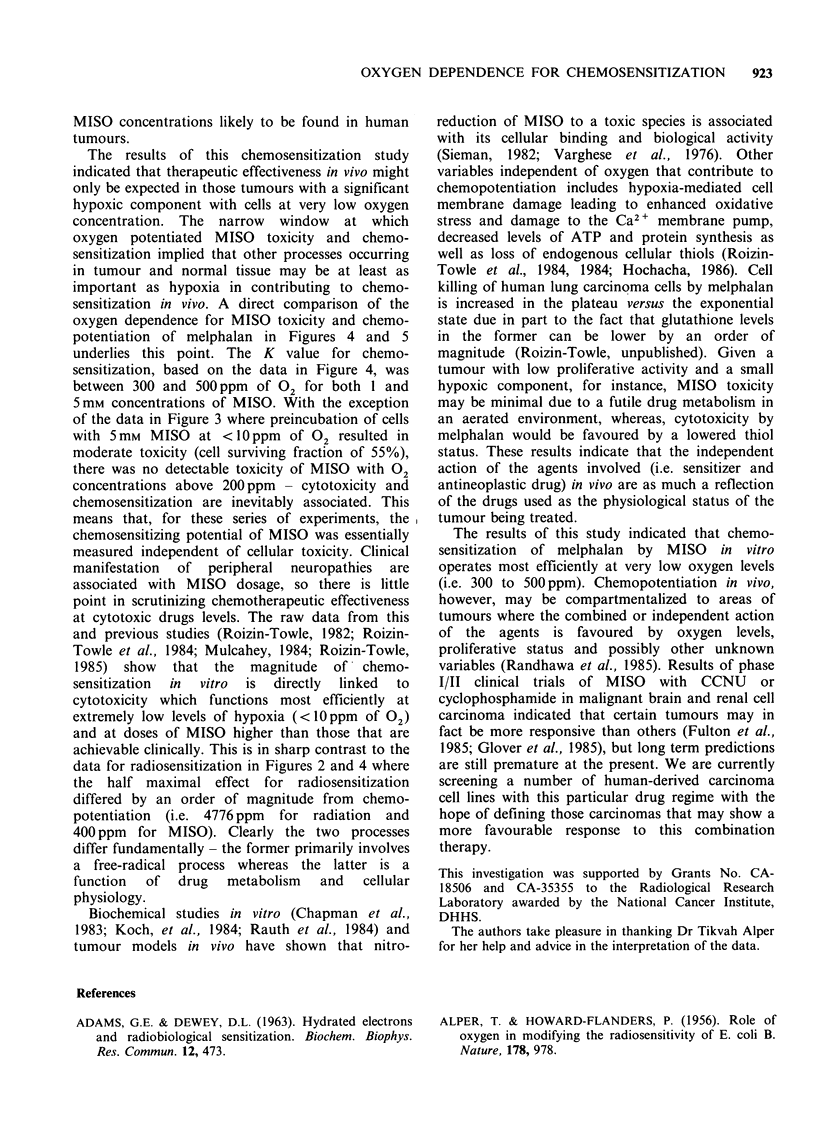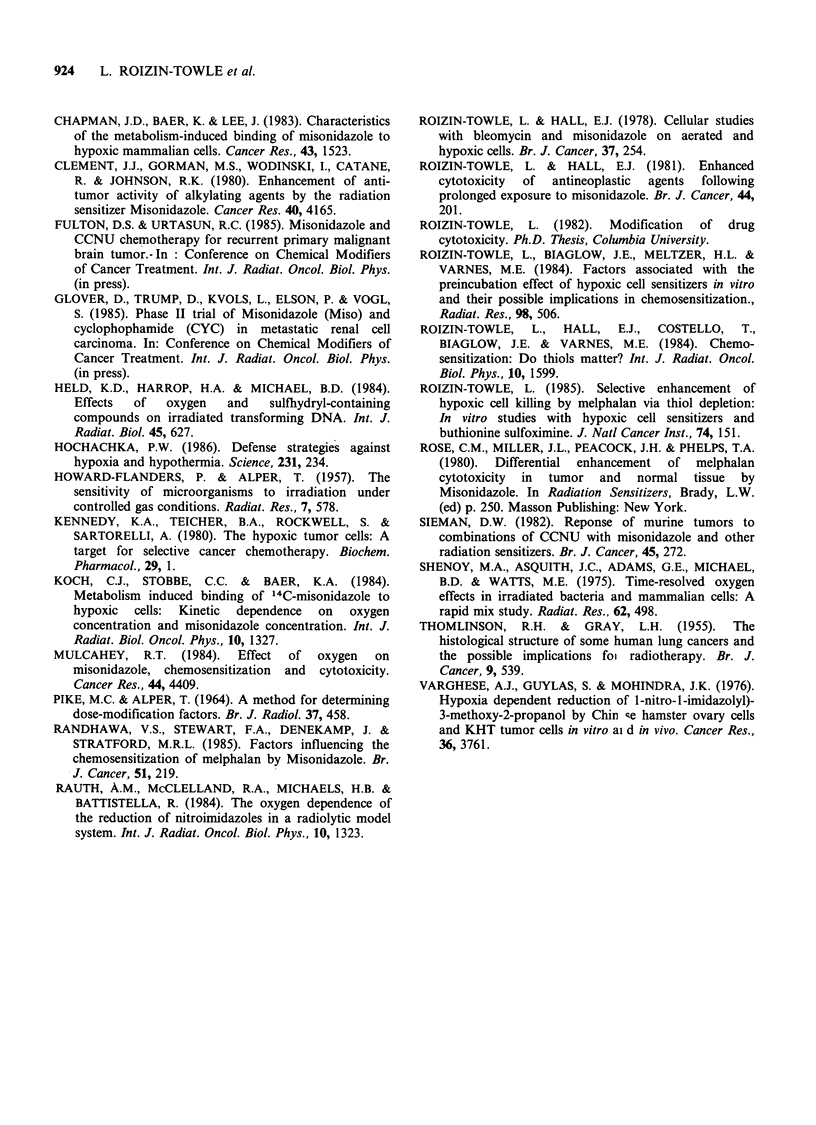# Oxygen dependence for chemosensitization by misonidazole.

**DOI:** 10.1038/bjc.1986.262

**Published:** 1986-12

**Authors:** L. Roizin-Towle, E. J. Hall, J. P. Pirro

## Abstract

Misonidazole (MISO) potentiates the cell killing effect of certain chemotherapy agents, but only under hypoxic conditions. The purpose of the present study was to define the range of oxygen concentrations over which chemosensitization by MISO takes place using mammalian cells cultured in vitro, and to compare this with the oxygen levels required for radiosensitization. V-79 hamster cells, attached to permanox dishes, were gassed with known concentrations of oxygen (less than 10 to 200,000 ppm) and treated with 1 and 5 mM MISO for 4 h previous to exposure to the chemotherapy agent, melphalan. In a parallel series of experiments, under the same gassing conditions, cells were irradiated with graded doses of X-rays at various oxygen concentrations. The K factor i.e. the oxygen concentration which defined half the maximum effect was found to be approximately 4776 ppm for radiosensitization and approximately 400 ppm for chemosensitization by MISO. It is evident that a significantly more stringent level of hypoxia is required for chemosensitization by MISO to take place than for radiosensitization.


*Br. J. Cancer* (1986), **54**, 919–924

### Oxygen dependence for chemosensitization by misonidazole

L. Roizin-Towle, E.J. Hall & J.P. Pirro

*Radiological Research Laboratory, College of Physicians and Surgeons, Columbia University, New York, NY 10032, USA.*

**Summary** Misonidazole (MISO) potentiates the cell killing effect of certain chemotherapy agents, but only under hypoxic conditions. The purpose of the present study was to define the range of oxygen concentrations over which chemosensitization by MISO takes place using mammalian cells cultured *in vitro,* and to compare this with the oxygen levels required for radiosensitization. V-79 hamster cells, attached to permanox dishes, were gassed with known concentrations of oxygen (<10 to 200,000 ppm) and treated with 1 and 5 mM MISO for 4 h previous to exposure to the chemotherapy agent, melphalan. In a parallel series of experiments, under the same gassing conditions, cells were irradiated with graded doses of X-rays at various oxygen concentrations. The *K* factor i.e. the oxygen concentration which defined half the maximum effect was found to be ~4776 ppm for radiosensitization and ~400 ppm for chemosensitization by MISO. It is evident that a significantly more stringent level of hypoxia is required for chemosensitization by MISO to take place than for radiosensitization.

The effect of molecular oxygen on the sensitivity of cells and tissues to radiation has been of major interest to radiobiologists and radiation therapists alike, since its importance was fully realized several decades ago by Thomlinson and Gray (1955). In transplanted tumours in small rodents, exposed to single large doses of radiation, tumour control is certainly governed by the presence of *foci* of chronically hypoxic cells which are intransigent to killing by X-rays and constitute a focus for the regrowth of the tumour. To what extent the same is true in human tumours, treated with multiple small doses of radiation, has been the subject of speculation for many years. A number of innovative strategies have been introduced in an attempt to decrease the differential radiosensitivity between oxygenated and hypoxic cells. The first of these was the introduction of hyperbarbic oxygen in an attempt to cause oxygen to diffuse to regions otherwise hypoxic; an alternative effort was the use of more densely inonizing radiations for which the presence or absence of molecular oxygen has a smaller influence on the degree of cell kill; the most recent attempt is the development of sensitizers that are electron affinic and mimic oxygen and thus sensitize hypoxic cells without having any significant influence on aerated cells (Adams & Dewey, 1963).

The realization that hypoxia may have an influence on the success of chemotherapy is of much more recent date (Roizin-Towle & Hall, 1978; Kennedy *et al.*, 1980). Sensitizers developed initially to increase the sensitivity of hypoxic cells to radiation have been found also to increase the sensitivity of cells to chemotherapy agents (Roizin-Towle & Hall, 1981). Chemosensitization however, requires that the cells be exposed for prolonged periods of time to the sensitizing drugs, and at least *in vitro* hypoxia appears to be an absolute requirement. In transplantable tumours in small laboratory rodents, sensitization of cells to alkylating agents by the use of misonidazole has a magnitude which appears to be much greater than can be accounted for on the basis of the known proportion of radiobiologically hypoxic cells. The realization that selective killing of hypoxic cells by MISO may not be the sole factor in chemosensitization was suggested by earlier studies *in vivo* (Sieman, 1982; Rose *et al.*, 1980; Clement *et al.*, 1980). The experiments reported in this paper were designed to investigate the role of oxygen in chemosensitization and to compare on an equal basis the *K* factor, that is the concentration of oxygen characteristic of a sensitivity half way between complete anoxia and full oxygenation, for chemosensitization and radiosensitization.

Correspondence: L. Roizin-Towle
Received 21 April 1986; and in revised form 8 August 1986.

### Materials and methods

V-79 hamster fibroblast cells in exponential phase of growth were used for these experiments. The cells were maintained in F-10 medium, supplemented with 10% foetal calf serum, penicillin/streptomycin and 1% glutamine. Cells were plated onto permanox dishes (Lux Corp.) the day before an experiment at numbers estimated to give 100–200 surviving colonies per treatment dose.

© The Macmillan Press Ltd., 1986

The equipment used was designed and fabricated in our own machine shop. Sensitization of cells by MISO to lethality by melphalan, and sensitization of V-79 cells to 30 kVp X-rays was studied as a function of varying concentrations of oxygen. In both series of experiments, (chemosensitization or radiosensitization) the cells were treated under identical conditions so that the *K* curves could be compared directly. The advantages of this system over other techniques (such as spinner culture) are that cells remain attached throughout the entire procedure, avoiding clumping and mechanical shearing of the cells by a stirring apparatus. It also avoids several centrifugation procedures needed to remove sensitizing drugs which results in cell loss. The nature of the 'preincubation' studies require extended periods of time, and this system allows not only sufficient time for equilibrium of gas with medium, but allows cells to be irradiated or treated with drug at a minimum of disturbance. Cells were plated at a density of 200 to 5,000 per dish depending on the expected level of survival.

The apparatus consisted of two rectangular boxes, each of which could accommodate 16 permanox culture dishes (Miles Labs., Naperville, Illinois). The containment boxes were connected to each other by Tygon tubing and were further connected by standard fittings to certified gas tanks. Gas was humidified before flowing through the lucite boxes, and a water trap was connected at the exhaust end of the system to prevent gas flowback. A Thermox 1A Oxygen Analyzer (Ametex, Pittsburg, Pennsylvania) was used to check the oxygen content of the gas tanks and to measure the $O_2$ content of the gas effluent after its route through the apparatus. The flow-rate was 8 l min$^{-1}$, compared with a total volume of the containers of 0.1 l.

For any given experiment, dishes containing cells were placed in the lucite boxes on a metal frame attached to a mechanical linkage that rocked the boxes at 6 rpm in a 37.5°C water bath. This was to ensure gas equilibration with the 3 ml medium overlying the cells. To speed equilibration, the lucite boxes were alternately gassed and evacuated for 1 h. Once the desired level of hypoxia was achieved, the rocking was discontinued, but the gas flow was maintained through the prolonged periods of preincubation with MISO.

For the chemosensitization experiments, half the dishes contained either 1 or 5 mM MISO, while the others served as the untreated control. Cells were pretreated at a given $O_2$ concentration, with or without MISO, for a total of 4 h at 37.5°. After the preincubation period four untreated control dishes were given a test dose of X-rays, and the resulting surviving fraction compared with previous radiosensitization experiments to serve as an internal check that there were no air leaks in the system. Following the preincubation treatment, the lucite boxes containing the dishes were removed from the water bath, the cells were washed free of MISO and a 1 h exposure of cells to graded doses of L-PAM at 37.5°C begun. At the completion of this drug exposure, the dishes were once again washed free of drug, replenished with fresh medium, and incubated for 7 days at 37.5°C to allow for colony formation. The MISO cytotoxicity in each preincubation exposure, and MISO enhancement ratio for melphalan killing were calculated from the survival curves generated by each experiment. A whole series of experiments were performed for $O_2$ concentrations ranging from $<10$ to 200,000 ppm $O_2$. $D_0$s for control and MISO pretreated cells were used to estimate the MISO sensitivity ratio, and ultimately the *K* factor for the preincubation effect.

Radiosensitization experiments involved the same basic procedure except that at the end of the gassing period, the dishes were irradiated *in situ* with the gas mixture still flowing, and no MISO was used in the pretreatment. An aerated survival curve was performed in each experiment and the OER and the sensitization factor evaluated each time. Irradiations were performed with a Sieman's Stabilipan X-ray therapy machine, operating at 300 kVp 30 mA with added filtration of 0.2 mm copper and 0.5 mm aluminium. The dose rate at the position occupied by the cells was computed to be 3.6 Gy min$^{-1}$. Survival curves generated by exposure of cells to a whole range of $O_2$ concentrations produced data necessary to calculate the *K* value for radiosensitization.

### Results

Data from a representative experiment illustrating the dependence of radiosensitivity on oxygen concentration are shown in Figure 1; the series of dose response curves were derived for cells irradiated under various oxygen concentrations using the permanox dishes and apparatus described above. A series of survival curves were generated in this fashion with relative sensitivity defined as the inverse of the $D_0$, where $D_0$ is calculated in the usual way from the slopes of the exponential tails of the shouldered survival curves using the Pike–Alper computer fit (Pike & Alper, 1964). Evaluation of all test oxygen concentrations in one day was impossible so that data points in Figure 2 represent the accumulated results of several experiments where data was eliminated unless it was within 95% confidence limits.

**Figure 1** Radiation survival curves of V-79 cells as a function of oxygen concentration.

**Figure 2** Relative sensitivity as a function of oxygen concentration for cells treated with 300 kVp X-rays. The $K$ value of this curve plotted on a linear-linear plot was 4776 ppm ± 100 ppm $O_2$.

Data are plotted on a linear-linear scale in a similar fashion to that used by others in documenting this effect (Howard-Flanders & Alper, 1957; Held *et al.*, 1984; Shenoy *et al.*, 1975). Data were fitted to an equation relating a given concentration of oxygen to relative radiosensitivity based on a formula by Alper and Howard-Flanders (1956) as shown in Equation 1.

$$r=(m[O_2]+K)/([O_2]+K) \quad (1)$$

It was semi-empirically derived by Alper and Howard-Flanders from their equation relating a given partial pressure of oxygen to the extent of radiosensitization in bacterial cells where $r$ was the sensitivity at a particular concentration or particular pressure of oxygen $[O_2]$, relative to that under anoxia and $m$ was the OER or oxygen enhancement ratio. Sensitivity ($r$) was defined as the inverse of the dose required to give an average of one lethal event per cell ($D_0^{-1}$), $D_0$ being calculated in the usual way from the slope of the exponential tails of the shouldered survival curves. The term we used to represent radiosensitivity ($r$) was proportional to the inverse of the $D_0$ of the survival curve and was assigned a value of 1 for anoxia and a maximum $r$ value of 3.7 for the maximum aerated response. The break in the survival curve between 5,000 and 200,000 ppm $O_2$ is shown this way as estimations of $r$ were not made at these oxygen levels.

Howard-Flanders and Alper both showed that Equation 1 could be derived theoretically and may be rewritten as:

$$(r-1)/(m-r)=[O_2]/K. \quad (2)$$

Our radiation data were well fitted by the equations given above. A $K$ value, or sensitivity half way between anoxia and air, occurred at a concentration of about 4776 ± 100 ppm or 0.47% of oxygen. This value was similar to other values for mammalian cells cited in the literature. The enhancement ratio for chemosensitization was calculated as the ratio of drug dosages of control to preincubated cells that produced a survival level of 10%. The standard errors on the points shown in Figure 2 represent pooling of data at the same concentration for several repeat experiments.

Figure 3 illustrates that chemosensitization of hamster cells by MISO is at a maximum when oxygen levels are < 10 ppm. This figure shows pooled data from two experiments demonstrating the sensitizing effect of MISO (1 and 5 mM) on cellular toxicity to melphalan. Chemosensitization of cells by MISO in contrast to radiosensitization, is defined by very low levels of oxygen.

Pretreatment of cells at a range of oxygen concentrations generated a series of survival curves which were computer-filled to the Pike–Alper equation that was also used to fit the radiation survival curves. We defined a MISO sensitivity ratio ($r$) in a fashion similar to the way we calculated $r$ for radiosensitivity. A series of survival curves derived from cells exposed to melphalan were generated at a wide range of $[O_2]$ concentrations as illustrated in Figure 3. $D_0$s were calculated from each MISO-pretreated and control curve and $r$ determined according to the equation below:

$$\frac{D_0^{-1}[\text{MISO}+\text{L-PAM}]}{D_0^{-1}[\text{L-PAM}]}=r. \quad (4)$$

**Figure 3** Chemosensitization of melphalan-treated cells by preincubation with two doses of MISO (1 and 5 mM) at a gas concentration of $<10$ ppm $O_2$.

Figure 4 summarizes the results of these pooled experiments where a ratio of 1 indicated no effect and 5 was the maximum enhancement ratio seen with preincubation of cells with 5 mM MISO. These ratios were corrected for cytotoxicity, if any, in the preincubation exposures. Calculations of $K$ factors for chemosensitization of MISO using Equations 2 and 3 did not yield the values we estimated experimentally from the data in Figure 4. Estimating the $K$ factor as that value which yields half the maximal effect (i.e. $(m+1)/2$ yielded a $K$ value between 300 and 500 ppm of $O_2$. This is a reasonably good estimate for the oxygen concentration required to reduce chemosensitization by 1/2 given the stringent conditions and care with which these experiments were performed. The close association between cytotoxicity in preincubation exposures and chemosensitization is illustrated in Figure 5, where cytotoxicity of 1 and 5 mM MISO is looked at as a function of oxygen concentration. Measurable cytotoxicity with these drug pretreatments was only seen at oxygen levels below 200 ppm. No cytotoxicity was seen for 1 mM MISO at values of $O_2$ of 200 ppm and above, and only 10% for the 5 mM pretreatment dose. This implies that half the chemosensitization effect is still functional at levels of MISO that are non-cytotoxic, although the drug enhancement factors are substantially reduced especially in the case of 1 mM MISO (i.e. sensitivity ratio of 1.4) which is most comparable to drug dosages achievable in a clinical situation.

### Discussion

The combined use of an hypoxic cell sensitizer with a cancer therapeutic agent to enhance the killing of hypoxic cells resistant to antineoplastic agents is a relatively recent idea (Roizin-Towle & Hall, 1978; Rose *et al.*, 1980), but one which has questioned the importance of oxygen as a modifier of drug toxicity. Focussing on the importance of oxygen the $K$ factor for MISO chemosensitization of melphalan and the $K$ factor for radiosensitization were compared on an equal basis. The objective was to determine whether chemosensitization was functional at intermediate levels of oxygen, and at

**Figure 4** Oxygen dependence of chemosensitization of cells by MISO illustrating the narrow range of oxygen levels (300–500 ppm $O_2$) at which half the pre-incubation effect is lost.

**Figure 5** The cytotoxicity of MISO (1 and 5 mM) as a function of oxygen concentration.

MISO concentrations likely to be found in human tumours.

The results of this chemosensitization study indicated that therapeutic effectiveness *in vivo* might only be expected in those tumours with a significant hypoxic component with cells at very low oxygen concentration. The narrow window at which oxygen potentiated MISO toxicity and chemosensitization implied that other processes occurring in tumour and normal tissue may be at least as important as hypoxia in contributing to chemosensitization *in vivo*. A direct comparison of the oxygen dependence for MISO toxicity and chemopotentiation of melphalan in Figures 4 and 5 underlies this point. The $K$ value for chemosensitization, based on the data in Figure 4, was between 300 and 500 ppm of $O_2$ for both 1 and 5 mM concentrations of MISO. With the exception of the data in Figure 3 where preincubation of cells with 5 mM MISO at $<10$ ppm of $O_2$ resulted in moderate toxicity (cell surviving fraction of 55%), there was no detectable toxicity of MISO with $O_2$ concentrations above 200 ppm – cytotoxicity and chemosensitization are inevitably associated. This means that, for these series of experiments, the chemosensitizing potential of MISO was essentially measured independent of cellular toxicity. Clinical manifestation of peripheral neuropathies are associated with MISO dosage, so there is little point in scrutinizing chemotherapeutic effectiveness at cytotoxic drugs levels. The raw data from this and previous studies (Roizin-Towle, 1982; Roizin-Towle *et al.*, 1984; Mulcahey, 1984; Roizin-Towle, 1985) show that the magnitude of chemosensitization *in vitro* is directly linked to cytotoxicity which functions most efficiently at extremely low levels of hypoxia ($<10$ ppm of $O_2$) and at doses of MISO higher than those that are achievable clinically. This is in sharp contrast to the data for radiosensitization in Figures 2 and 4 where the half maximal effect for radiosensitization differed by an order of magnitude from chemopotentiation (i.e. 4776 ppm for radiation and 400 ppm for MISO). Clearly the two processes differ fundamentally – the former primarily involves a free-radical process whereas the latter is a function of drug metabolism and cellular physiology.

Biochemical studies *in vitro* (Chapman *et al.*, 1983; Koch, *et al.*, 1984; Rauth *et al.*, 1984) and tumour models *in vivo* have shown that nitroreduction of MISO to a toxic species is associated with its cellular binding and biological activity (Sieman, 1982; Varghese *et al.*, 1976). Other variables independent of oxygen that contribute to chemopotentiation includes hypoxia-mediated cell membrane damage leading to enhanced oxidative stress and damage to the $Ca^{2+}$ membrane pump, decreased levels of ATP and protein synthesis as well as loss of endogenous cellular thiols (Roizin-Towle *et al.*, 1984, 1984; Hochacha, 1986). Cell killing of human lung carcinoma cells by melphalan is increased in the plateau *versus* the exponential state due in part to the fact that glutathione levels in the former can be lower by an order of magnitude (Roizin-Towle, unpublished). Given a tumour with low proliferative activity and a small hypoxic component, for instance, MISO toxicity may be minimal due to a futile drug metabolism in an aerated environment, whereas, cytotoxicity by melphalan would be favoured by a lowered thiol status. These results indicate that the independent action of the agents involved (i.e. sensitizer and antineoplastic drug) *in vivo* are as much a reflection of the drugs used as the physiological status of the tumour being treated.

The results of this study indicated that chemosensitization of melphalan by MISO *in vitro* operates most efficiently at very low oxygen levels (i.e. 300 to 500 ppm). Chemopotentiation *in vivo*, however, may be compartmentalized to areas of tumours where the combined or independent action of the agents is favoured by oxygen levels, proliferative status and possibly other unknown variables (Randhawa *et al.*, 1985). Results of phase I/II clinical trials of MISO with CCNU or cyclophosphamide in malignant brain and renal cell carcinoma indicated that certain tumours may in fact be more responsive than others (Fulton *et al.*, 1985; Glover *et al.*, 1985), but long term predictions are still premature at the present. We are currently screening a number of human-derived carcinoma cell lines with this particular drug regime with the hope of defining those carcinomas that may show a more favourable response to this combination therapy.

This investigation was supported by Grants No. CA-18506 and CA-35355 to the Radiological Research Laboratory awarded by the National Cancer Institute, DHHS.

The authors take pleasure in thanking Dr Tikvah Alper for her help and advice in the interpretation of the data.

### References

ADAMS, G.E. & DEWEY, D.L. (1963). Hydrated electrons and radiobiological sensitization. *Biochem. Biophys. Res. Commun.* **12**, 473.

ALPER, T. & HOWARD-FLANDERS, P. (1956). Role of oxygen in modifying the radiosensitivity of E. coli B. *Nature*, **178**, 978.

CHAPMAN, J.D., BAER, K. & LEE, J. (1983). Characteristics of the metabolism-induced binding of misonidazole to hypoxic mammalian cells. *Cancer Res.*, **43**, 1523.

CLEMENT, J.J., GORMAN, M.S., WODINSKI, I., CATANE, R. & JOHNSON, R.K. (1980). Enhancement of antitumor activity of alkylating agents by the radiation sensitizer Misonidazole. *Cancer Res.* **40**, 4165.

FULTON, D.S. & URTASUN, R.C. (1985). Misonidazole and CCNU chemotherapy for recurrent primary malignant brain tumor.- In : Conference on Chemical Modifiers of Cancer Treatment. *Int. J. Radiat. Oncol. Biol. Phys.* (in press).

GLOVER, D., TRUMP, D., KVOLS, L., ELSON, P. & VOGL, S. (1985). Phase II trial of Misonidazole (Miso) and cyclophophamide (CYC) in metastatic renal cell carcinoma. In: Conference on Chemical Modifiers of Cancer Treatment. *Int. J. Radiat. Oncol. Biol. Phys.* (in press).

HELD, K.D., HARROP, H.A. & MICHAEL, B.D. (1984). Effects of oxygen and sulfhydryl-containing compounds on irradiated transforming DNA. *Int. J. Radiat. Biol.* **45**, 627.

HOCHACHKA, P.W. (1986). Defense strategies against hypoxia and hypothermia. *Science*, **231**, 234.

HOWARD-FLANDERS, P. & ALPER, T. (1957). The sensitivity of microorganisms to irradiation under controlled gas conditions. *Radiat. Res.*, **7**, 578.

KENNEDY, K.A., TEICHER, B.A., ROCKWELL, S. & SARTORELLI, A. (1980). The hypoxic tumor cells: A target for selective cancer chemotherapy. *Biochem. Pharmacol.*, **29**, 1.

KOCH, C.J., STOBBE, C.C. & BAER, K.A. (1984). Metabolism induced binding of $^{14}$C-misonidazole to hypoxic cells: Kinetic dependence on oxygen concentration and misonidazole concentration. *Int. J. Radiat. Biol. Oncol. Phys.*, **10**, 1327.

MULCAHEY, R.T. (1984). Effect of oxygen on misonidazole, chemosensitization and cytotoxicity. *Cancer Res.*, **44**, 4409.

PIKE, M.C. & ALPER, T. (1964). A method for determining dose-modification factors. *Br. J. Radiol.* **37**, 458.

RANDHAWA, V.S., STEWART, F.A., DENEKAMP, J. & STRATFORD, M.R.L. (1985). Factors influencing the chemosensitization of melphalan by Misonidazole. *Br. J. Cancer*, **51**, 219.

RAUTH, A.M., McCLELLAND, R.A., MICHAELS, H.B. & BATTISTELLA, R. (1984). The oxygen dependence of the reduction of nitroimidazoles in a radiolytic model system. *Int. J. Radiat. Oncol. Biol. Phys.*, **10**, 1323.

ROIZIN-TOWLE, L. & HALL, E.J. (1978). Cellular studies with bleomycin and misonidazole on aerated and hypoxic cells. *Br. J. Cancer*, **37**, 254.

ROIZIN-TOWLE, L. & HALL, E.J. (1981). Enhanced cytotoxicity of antineoplastic agents following prolonged exposure to misonidazole. *Br. J. Cancer*, **44**, 201.

ROIZIN-TOWLE, L. (1982). Modification of drug cytotoxicity. *Ph.D. Thesis, Columbia University*.

ROIZIN-TOWLE, L., BIAGLOW, J.E., MELTZER, H.L. & VARNES, M.E. (1984). Factors associated with the preincubation effect of hypoxic cell sensitizers *in vitro* and their possible implications in chemosensitization., *Radiat. Res.*, **98**, 506.

ROIZIN-TOWLE, L., HALL, E.J., COSTELLO, T., BIAGLOW, J.E. & VARNES, M.E. (1984). Chemosensitization: Do thiols matter? *Int. J. Radiat. Oncol. Biol. Phys.*, **10**, 1599.

ROIZIN-TOWLE, L. (1985). Selective enhancement of hypoxic cell killing by melphalan via thiol depletion: *In vitro* studies with hypoxic cell sensitizers and buthionine sulfoximine. *J. Natl Cancer Inst.*, **74**, 151.

ROSE, C.M., MILLER, J.L., PEACOCK, J.H. & PHELPS, T.A. (1980). Differential enhancement of melphalan cytotoxicity in tumor and normal tissue by Misonidazole. In *Radiation Sensitizers*, Brady, L.W. (ed) p. 250. Masson Publishing: New York.

SIEMAN, D.W. (1982). Reponse of murine tumors to combinations of CCNU with misonidazole and other radiation sensitizers. *Br. J. Cancer*, **45**, 272.

SHENOY, M.A., ASQUITH, J.C., ADAMS, G.E., MICHAEL, B.D. & WATTS, M.E. (1975). Time-resolved oxygen effects in irradiated bacteria and mammalian cells: A rapid mix study. *Radiat. Res.*, **62**, 498.

THOMLINSON, R.H. & GRAY, L.H. (1955). The histological structure of some human lung cancers and the possible implications foı radiotherapy. *Br. J. Cancer*, **9**, 539.

VARGHESE, A.J., GUYLAS, S. & MOHINDRA, J.K. (1976). Hypoxia dependent reduction of 1-nitro-1-imidazolyl)-3-methoxy-2-propanol by Chin se hamster ovary cells and KHT tumor cells *in vitro* aı d *in vivo*. *Cancer Res.*, **36**, 3761.